# Intrauterine Contraceptive Device Migrated in the Urinary Tract: Case Report and Extensive Literature Review

**DOI:** 10.3390/jcm13144233

**Published:** 2024-07-19

**Authors:** Valentin Nicolae Varlas, Andreea Ioana Meianu, Andra Ioana Rădoi, Irina Balescu, Nicolae Bacalbasa, Roxana Georgiana Varlas

**Affiliations:** 1 Department of Obstetrics and Gynecology, Carol Davila University of Medicine and Pharmacy, 020021 Bucharest, Romania; valentin.varlas@umfcd.ro; 2 Department of Obstetrics and Gynecology, Filantropia Clinical Hospital, 011132 Bucharest, Romania; meianu.andreea@gmail.com (A.I.M.); andra-ioana.radoi@rez.umfcd.ro (A.I.R.); 3 Doctoral School, “Carol Davila” University of Medicine and Pharmacy, 050474 Bucharest, Romania; roxana-georgiana.bors@drd.umfcd.ro; 4 Department of Surgery, “Carol Davila” University of Medicine and Pharmacy, 020021 Bucharest, Romania; 5 Department of Visceral Surgery, Center of Excellence in Translational Medicine “Fundeni” Clinical Institute, 022328 Bucharest, Romania

**Keywords:** intrauterine device, translocated, migration, intraperitoneally, embedment, bladder, ureter, urinary tract

## Abstract

The migration or translocation of an intrauterine device (IUD) in the urinary tract is a rare event. Here, we present the case of a 55-year-old woman who accidentally discovered the ectopic presence of an IUD following a radiological examination for pelvic pain caused by a lumbar discopathy. Over the years, the patient had several IUDs inserted without being able to specify which one had migrated. The removal of the IUD was performed laparoscopically with the minimum resection of the bladder wall and the subsequent cystorrhaphy. The evolution of the patient was favorable. To better analyze these events, we conducted an all-time extensive electronic search of the PubMed database and identified 94 eligible articles, with a total of 115 cases. The literature analysis on the IUD migrations shows either the simultaneous existence of the second IUD or of a maximum number of up to two IUD insertions during the life of patients. Thus, in the presented case, we identified five IUD insertions over time, which explained the chronic inflammatory process by forming an important mass of adherents that included the urinary bladder, uterus, omentum, sigmoid colon, and abdominal wall. Therapeutic management must be adapted to each case depending on the intra/extravesical location of the migrated IUD evaluated by imaging.

## 1. Introduction

The intrauterine device (IUD) is one of the most widely used long-term contraceptive methods in the world. This device is effective, long-acting, and reversible and can be used by many women [1]. It is known that 23% of those who use contraceptive methods have IUDs. The IUD’s popularity is probably due to its similar effectiveness to surgical sterilization and its safety and reversible effect. There are many mechanisms of action for the IUD, which vary by type of IUD (inert, copper, or hormonal) [2]. The most popular types of IUDs are copper T-shaped ones and those that release levonorgestrel, a progestin with similar contraceptive effectiveness to combined oral contraceptives when used correctly.

In contrast, IUDs are safer than improperly used oral contraceptives. Once inserted into the uterus, the IUD leads to a sterile inflammatory response that is unfavorable to sperm and eggs. Progestin-based IUDs have the added benefit of causing a thickening of the cervical mucus, making it more difficult for the sperm to reach the egg [3]. Progestins can cause endometrial decidualization and glandular atrophy, preventing implantation [2]. Almost 35% of patients may experience amenorrhea after 2 years of use. For patients with a history of deep vein thrombosis, pulmonary embolism, or myocardial infarction, the copper IUD is the first-line contraceptive method, thus avoiding hormonal treatment and its prothrombotic effects [3,4]. Among those using IUDs, there is an average rate of about six pregnancies per 1000 women, the failure rate being approximately 0.8% for the copper-containing IUD and 0.2% for the levonorgestrel-releasing IUD [5].

The most common side effects of the IUD are pain and irregular bleeding, especially in the first several months after insertion. Rarely, the IUD can be complicated by pelvic inflammatory disease, contraceptive failure, expulsion, perforation, or migration [6].

Uterine perforation during insertion is rare, occurring in 0.6 to 16 cases per 1000 procedures. The risk of perforation is higher when it is inserted between 4 and 6 weeks after delivery or elective abortion. There are two types of uterine perforations, and both are prone to severe complications. Perforation usually occurs at the time of IUD insertion but rarely can occur later [7]. Uterine perforation after IUD insertion may be observed during device insertion, immediately after the procedure, or as a delayed event.

Primary perforation may occur during insertion and is usually associated with severe abdominal pain. Risk factors for perforation include inexperience of the person inserting the IUD, retroverted uterus, immobile uterus, and myometrial defect from a previous cesarean section or myomectomy [8,9]. Delayed perforation may be due to uterine spasms, and it can be due to gradual pressure necrosis of the uterine wall [7]. Approximately 80% of IUDs migrate into the peritoneal cavity after perforation. Migration into surrounding organs is a rare but severe complication [10,11]. After perforation, IUDs can migrate to any location, including the omentum, pouch of Douglas, or adherent to the sigmoid colon. Less commonly, IUDs can migrate into the bladder, small intestine, appendix, or colon [1].

About 30% of those with uterine perforation are asymptomatic, while about 70% have abdominal pain or uterine bleeding [12]. The most common finding associated with IUD migration is “missing strings” [13]. The effectiveness of the IUD is maintained only if the device is correctly placed in the uterine cavity; therefore, it is important to teach the patient how to check the presence of the IUD strings at the first visit [14]. Long-term complications include the formation of abscesses and fistulas. The copper IUD causes a greater inflammatory process than the progesterone-based ones [13].

If the IUD is not found, different methods can be used to recover the device. The World Health Organization recommends removing the migrated device as soon as possible [15]. Surgical removal is recommended even in asymptomatic patients once the IUD has migrated outside the uterus. The recommendation is to use minimally invasive methods as far as possible. These include hysteroscopy, cystoscopy, gastrointestinal tract endoscopy, or laparoscopy, depending on where the IUD has migrated [16].

If the strings are not seen or felt on examination, it is important to proceed with transvaginal ultrasound or other imaging techniques before assuming that the IUD has been expelled through the cervix and vagina. If migration is identified, the next step would be to obtain a cross-sectional image with CT or MRI to evaluate the involvement of other organs. If the device is embedded in an organ, for example, the bladder or bowel, it is not recommended to remove it using minimally invasive methods; instead, exploratory laparoscopy or even laparotomy should be performed. If the IUD is suspected to be embedded in a vessel or cannot be accurately visualized, a multidisciplinary approach is necessary, and collaboration between experienced surgeons is mandatory [15,16,17].

We aimed to present a case of IUD migration in the bladder wall and an all-time extensive literature review regarding these cases’ diagnosis and therapeutic management.

## 2. Case Presentation

A 55-year-old woman presented to our hospital, having been referred by a radiology service with the diagnosis of an IUD migrated outside the uterine cavity. The discovery was accidental following a radiological evaluation to investigate pelvic pain caused by a lumbar discopathy. During radiography, a radiopaque image was visualized in the hypogastrium, suggesting an extrauterine migrated IUD intraperitoneally (lateral deviated to the left). The patient is currently in menopause and has had two natural births and two miscarriages. She also has a history of appendectomy and glaucoma. The patient stated that the IUD was removed by another gynecologist 2 years ago, being surprised by the result of the X-ray and the fact that she was asymptomatic. The patient specified that she was not instructed to self-check her IUD strings.

Later, she attended a vaginal examination, during which the IUD strings were not visualized. The 2D/3D transvaginal and abdominal ultrasound scan did not show any evidence of the presence of an IUD inside the uterus. Also, the uterus shows two intramural uterine leiomyomas, located posteriorly, measuring 3.4 × 3.2 cm and 3.3 × 3.7 cm, respectively. Subsequent CT of the abdomen and pelvis revealed a T-shaped IUD-like structure that migrated anterior and superior to the uterine fundus region, positioned slightly obliquely to the right. Urinalysis and urine culture were normal.

**Figure 1:**
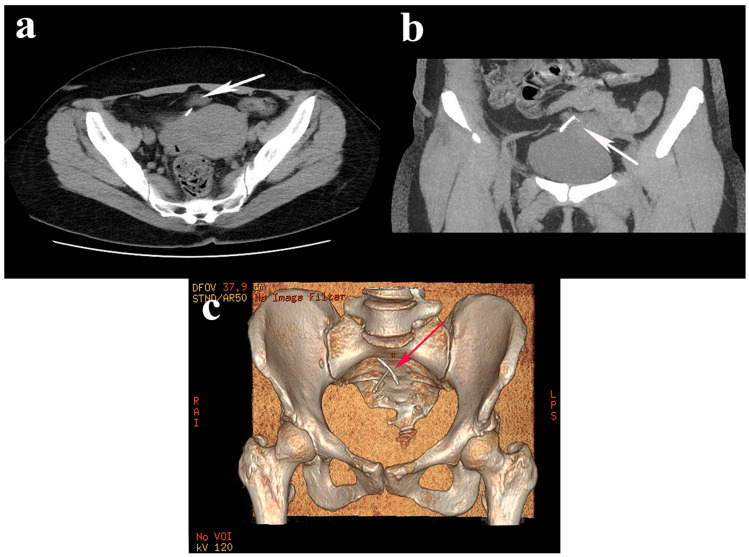
Axial (**a**) and sagittal (**b**) abdominopelvic computed tomography images revealed a translocated IUD above the bladder and uterus (white arrows), oriented slightly obliquely to the right; (**c**) 3D reconstruction of the IUD topography (red arrow).

**Figure 2:**
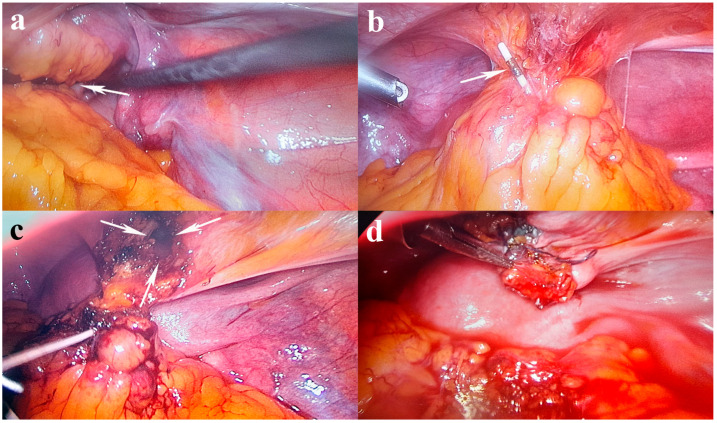
Laparoscopic findings: (**a**) pelvic mass (white arrow) with adhesions between the omentum, sigmoid colon, uterine fundus, and bladder; (**b**) embedded IUD in the pelvic mass (white arrow); (**c**) IUD removal from the mass with long arm penetrated in the bladder wall (white arrows show the bladder limited resection); (**d**) a double-layer cystorrhaphy with 2-0 wires.

A hysterolaparoscopy was proposed to specify the patient’s diagnosis. Diagnostic hysteroscopy revealed a regular cervical canal, a normal-looking uterine cavity, and a visible tubal ostia bilaterally. The surgical intervention was continued by performing a laparoscopy, which highlighted a pelvic mass with adhesions between the omentum, sigmoid colon, uterine fundus, and bladder. During the adhesiolysis, using the laparoscopic graspers, the limbs of the IUD intimately adherent to the serosa of the sigmoid colon and the long arm at the level of the submucosa of the urinary bladder could be identified. The dissection continued by traction of the IUD in its axis, with the IUD strings highlighted, without being able to detach the urinary bladder after sectioning the peritoneum over the bladder. The IUD was extracted en bloc to detach the strings with a limited resection of the bladder wall due to the technical difficulties created by the adhesions. A double-layer cystorrhaphy with 2-0 wires was performed, the associated adhesions were lysed, and the migrated IUD was successfully removed. The patient had a favorable evolution postoperatively and was discharged 48 h later. The evolution after one year was good, with the recommendation regarding the follow-up being regular medical check-ups (Figure 1 and Figure 2).

## 3. Discussion

The IUD is a globally accepted contraceptive method. IUDs are a popular method of reversible contraception because they have high efficacy, low risks, and relatively low cost. Post-insertion follow-up and awareness of complications are important to assess when to return to the gynecologist [18]. Expulsion, intrauterine displacement, translocation, and migration of the IUD lead to decreased contraceptive effectiveness and require immediate removal of the defective IUD with possible subsequent replacement to achieve contraception [1]. The first report on intravesical migration was made in 1972 by Rubin, who identified a device like the Lippes loop IUD [18].

Without a standardization of the management of these cases, we considered it necessary to conduct an all-time extensive literature search, accessing the PubMed database. Selection criteria were articles published in English and involving human subjects using the following methodological approaches: case series and case reports. The criteria followed were age, clinical manifestations, type of IUD, time of insertion, localization of IUD migration at the level of the urinary tract, size of the bladder stone, used imaging, therapeutic procedures, monitoring, and prognosis of these cases.

**Figure 3:**
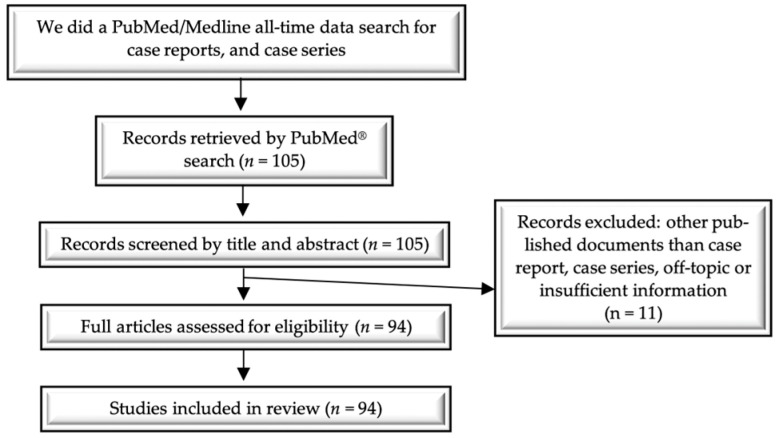
Flow diagram of the literature search and study selection process.

The key MeSH terms used in the electronic search technique were “intrauterine device”, “bladder”, “ureter”, “urinary tract”, “migration”, “translocation”, “embedment”, “diagnosis”, “treatment”, and “prognosis”. Finally, 105 articles were found, of which 94 met the eligibility criteria and were included in this review (Figure 3, Table 1).

**Figure 4:**
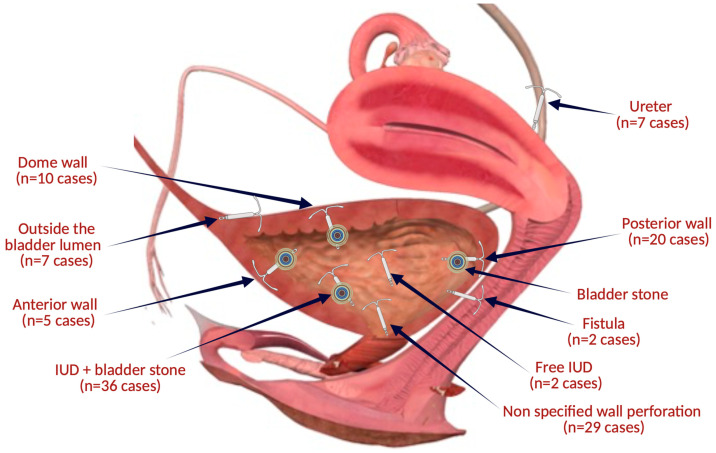
The topographical sites of IUD migrations related to the articles included in the review (the figure was made with the help of Biorender.com).

Thus, since 1980, 115 cases have been reported in which intrauterine contraceptive devices underwent a migration or translocation process with the interest of the urinary tract (Figure 4). More than 87 cases had bladder interest and 7 cases ureters. The average age of the patients in the review was 37.2 ± 10.71 years (range 19–74), with 11 cases >50 years. The average duration of IUD remaining in place after insertion reported in 96 cases was 8.4 ± 7.46 years (with an interval between 1 week and 35 years).

The main clinical findings encountered were the following: asymptomatic in 4.3% (*n* = 5), dysuria in 33.04% (*n* = 38), hematuria in 26.1% (*n* = 30), pelvic pain in 20% (*n* = 23), urinary tract infections (UTI) in 15.6% (*n* = 18), urinary tract symptoms (UTS) in 14.8% (n = 17), suprapubic pain in 11.3% (*n* = 13), abdominal pain in 6.1% (*n* = 7), and loin pain in 2.6% (*n* = 3). The imaging evaluation highlighted the use of US in 76 cases (66.1%), X-ray in 71 cases (61.7%), CT in 41 cases (35.6%), and IV urography in 13 cases (11.3%).

Regarding the therapeutic modalities, cystoscopy was recorded in 76 cases (66.1%), cystolithotomy in 11 cases (9.5%), cystotomy in 9 cases (7.8%), laparoscopy in 20 cases (17.4%) (1 case with Da Vinci), laparotomy in 9 cases (7.8%), cystolitholapaxy in 10 cases (8.7%), partial cystectomy in 1 case (0.87%), lithotripsy in 16 cases (13.8%) (including 2 cases with laser). The prognosis was favorable in 110 cases (95.6%), and complications (fistulas) were identified in 5 cases (4.34%).

To increase the quality of the study methodology and reduce bias in the case reports we analyzed in this review, we assessed articles using the criteria outlined by Murad et al. [111] (Table S1).

The incidence of uterine perforation secondary to the migration/translocation of an IUD is very low, ranging from 0.1 to 0.9% [35], but its real rate is unknown because many patients are asymptomatic, and discovery may be accidental during investigation for another possible pathology. The uterine perforation has an estimated incidence of 1.6 to 1000 inserts [4].

You should be especially concerned about perforation if the IUD has been inserted by an inexperienced healthcare provider or is placed in an inappropriate position [112]. Secondary perforation is a delayed event proposed to be due to gradual pressure necrosis of the uterine wall [7]. Another situation in which we might suspect a perforation is when the patient has a weakened uterine wall near the insertion site, which frequently occurs as a result of multiparity, cesarean section, or abortion [15]. Regarding risk factors, the patient presented in the above case gave birth twice vaginally, and she had two abortions.

Additional risk factors for perforation regarding the indication for IUD insertion can be found immediately postabortion or postpartum, and uterine malpositions (retroversion, post-cesarean scars). The transmigration of the inserted IUD is favored by uterine contractions that further push the IUD out of the intraperitoneal uterine wall or into adjacent areas such as the bladder, ureter, bowel, peritoneum, appendix, adnexa, and abdominal wall.

Spontaneous migration was not highlighted, but the mechanism was favored by the degree of penetration of the uterine wall at the time of insertion. The migration of the IUD into the urinary bladder can be associated with the formation of stones at the moment of penetration into the lumen of the urinary bladder, a process found in 65.2% (*n* = 75) of the studied cases. The longer the interval, the more the incidence of these bladder stones can increase.

The IUD’s postpartum insertion increases the risk of perforation due to the reduced consistency of the uterus, the reduced dimensions of the uterine wall, and the uterine contractions of increased intensity. Other factors, such as intestinal peristalsis and bladder muscle contractions, may promote progressive migration of the IUD. The moment when the IUD exceeds the mucosa and enters the bladder can be accompanied by certain clinical symptoms. Bjornerem et al. showed that about 2% of the IUD migrates involve the bladder [26].

Knowing the type and form of IUD is important because it is known that the copper IUD determines an important inflammatory process and must be removed. The prevention of IUD migration can be achieved by identifying risk factors and increasing doctors’ learning curves.

Many patients with uterine perforation and IUD migration may present with symptoms, but as many as 30% are asymptomatic [12], as in the presented case. The clinical findings of IUD migration can occur with wide variability (from one week to 35 years, according to Table 1) because some patients are asymptomatic, and others are not recognized as belonging to this situation. Studies show that even in patients who have been asymptomatic for a long time, the therapeutic removal of the IUD is necessary due to the possible complications that can occur and that increase the morbidity of these cases (abscess, fistulas, hydronephrosis, obstructions).

Patients at the time of presentation may have a series of clinical symptoms (recurrent UTIs, UTSs, urinary irritation, hematuria, incontinence, and abdominal pain) until the time of diagnosis and may be treated for long periods with antibiotics. The diagnosis of faulty placement of the IUD at the time of insertion can be indicated by bleeding, pain, or the lack of visualization of the strings in the vast majority of cases, but some occult perforations can be diagnosed after a variable period.

Transmigration of the IUD leads to the lack of contraceptive effect, which is why some patients became pregnant, and some gave birth at term. Extraction of the IUD is mandatory in the event of pregnancy or when the patient wishes to conceive due to the risk that may occur when it migrates. IUD left in situ during pregnancy predisposes to spontaneous abortions, placenta previa or abruption, preterm premature rupture of the membranes (PPROM), chorioamnionitis, or cesarean delivery [113].

IUDs are effective if they are used correctly, removed, and replaced at the right time. Most frequently, intraperitoneally migration occurs in patients with scarred uterus, but as in our presented case, it can also occur in those who have not undergone cesarean sections.

If a patient has a lost IUD and the strings are not visible during the pelvic exam, the correct diagnosis must be obtained before surgery, including transvaginal or abdominal ultrasound or even radiography to confirm the position of the IUD. If IUD migration is still suspected, cross-sectional imaging such as CT or MRI is recommended to rule out the involvement of adjacent organs before surgical removal [114,115].

We encounter diagnostic and therapeutic challenges in devices partially migrated into the bladder wall. The role of ultrasound is important when we suspect the existence of IUD migration due to the lack of intrauterine evidence of it or its presence in the adjacent structures. The imaging diagnosis of translocated IUDs can sometimes be performed by a single technique or by combinations thereof (US, X-ray, CT).

Cystoscopy in these cases is important for the intraluminal identification of the IUD at the level of the urinary bladder and therapeutic resolution where possible, either by pulling it or by using a forceps to crush the stones in case the IUD is embedded in the bladder calculus. Currently, minimally invasive endoscopic techniques prevail over open surgical interventions. The more extensive the involved area of the bladder wall, the higher the use rate of open surgical procedures that will repair the defect after IUD extraction.

In selected cases, in elderly patients with comorbidities who accidentally discovered the presence of a migrated IUD, we can either try a conservative treatment or postpone an intervention until we can perform it due to the increased risk of morbidity secondary to possible complications. Fortunately, few cases in the literature have been reported in menopausal women.

The World Health Organization recommends removing the migrated device immediately after it is detected. It is suggested that surgical removal should be considered even in asymptomatic patients once it has migrated outside the uterus, as it was in the case presented [116]. In most cases, the therapeutic solution was customized through minimally invasive combined techniques (cystoscopy ± hysteroscopy ± laparoscopy) depending on the intravesical or extravesical position of the IUD. Furthermore, these patients require a multidisciplinary approach (gynecologist, urologist) and a general surgeon’s presence in complicated cases. In the case of the presence of fistulas, they are solved exclusively by the urologist. Minimally invasive procedures, including hysteroscopy and laparoscopy, should be attempted first, as in our case. Since our patient had a history of deliveries and post-appendectomy adhesions, extra caution should be taken when exploring the abdomen for the migrated device. Locating the strings in the submucosa of the urinary bladder is a rare and potentially serious complication, but with careful adhesiolysis, the IUD removal was completed successfully.

In our case, an X-ray was performed first for another medical reason. After that, the asymptomatic patient underwent a vaginal examination, and the IUD strings were not visualized. CT was performed to identify the exact position of the migrated IUD. The migration of the IUD with the highlighting of the strings in the submucosal region of the urinary bladder and the adhesions of this pelvic mass with the omentum and the sigmoid colon represents a particularity of this case. Removing the IUD required adhesiolysis and limited resection of the bladder wall. A limitation of this study is the use of a single database search.

## 4. Conclusions

IUDs are effective and are frequently used as a contraceptive method, but patients must be instructed to self-check their IUD strings and return for periodic control to check the correct position of the IUD for contraceptive effectiveness and to avoid possible complications due to its migration. Early identification of IUD migration and its removal significantly lowers the complication rate. The removal of an IUD that has migrated to the level of the urinary tract is a major concern both in the case of patients with recurrent symptoms and in asymptomatic patients with an uncertain history regarding the inserted IUD. Therapeutic management must be adapted to each case depending on the intra/ extravesical location of the migrated IUD evaluated by imaging.

## Figures and Tables

**Table 1:** Synopsis of all-time literature studies regarding translocation/migration of IUD in the urinary tract.

Author, Year	Age	Clinical Findings	IUD Insertion Period	IUD Type	Stone Size	Imaging Technique	Site of IUD	Surgical Procedure	Follow-Up/ Prognosis
Woods [19], 1980	28	Dysuria, pelvic pain, hematuria	7 yrs	4	2.5	Excretory urogram, cystoscopy	The left side of the bladder fundus, partially embedded in the omentum	Laparotomy	Favorable
Sasidharan [20], 1988	47	Dysuria, frequency	14 yrs	1	NS	X-ray, IVU	Bladder stone	Cystoscopy—suprapubic cystolithotomy	Favorable
Khan [21], 1990	24	Missing strings	4 yrs	2	4.7	X-ray	Bladder stone	Cystoscopy	Favorable
Dietrick [22], 1992	38	Pelvic pressure, suprapubic pain, hematuria	16 yrs	4	5.2	X-ray, CT	Bladder stone	Cystoscopy—cystolithotomy	Favorable
Shratter [23], 1993	29	Pelvic pain	NS	2	NS	US	Bladder stone	Cystoscopy	Favorable
el-Diasty [24], 1993	40	Dysuria	8 yrs	1	4.5	X-ray, US	Bladder stone	Cystoscopy—cystolithotomy	Favorable
Caspi [25], 1996	35	Pelvic pain	3 yrs	2	NS	US	Partly on the bladder wall and bladder stone	Cystoscopy	Favorable
Bjørnerem [26], 1997	43	Pelvic pain, frequency, dysuria	3 wks	2	NS	US	Moving freely inside the bladder	Cystoscopy	Favorable
Szabó [27], 1997	30	Cyclical hematuria	4 yrs	NS	NS	Cystoscopy	Posterior bladder wall	Cystoscopy, laparotomy	Utero-vesical fistula after 6 wks
Maskey [28], 1997	19	Dysuria, frequency	7 yrs	2	NS	X-ray	Bladder stone	Suprapubic cystolithotomy	Favorable
	28	Frequency	6 yrs	2	NS	X-ray, US	Bladder stone	Suprapubic cystolithotomy	
Cumming [29], 1997	45	Frequency, stress incontinence	4 yrs	2	5	X-ray, urodynamic study	Posterior bladder wall and bladder stone	Cystoscopy—cystolitholapaxy	Favorable
Yalçin [30], 1998	35	Dysuria, frequency, suprapubic pain, urethral irritation	11 yrs	2	NS	US, X-ray, IVU revealed a stone	On top of the bladder	Cystoscopy	Favorable
Olaore [31], 1999	36	Asymptomatic	1 yr	1	NS	US	Partly on the bladder wall, with bladder stone	Cystoscopy	Favorable
Sehgal [32], 2000	33	Pelvic pain, hematuria	13 yrs	2	NS	X-ray, US	One limb in the bladder, the other one in the utero-vesical interface	Laparotomy—bladder repair	Favorable
Güvel [33], 2001	30	Dysuria, frequency, pelvic pain, hematuria	10 yrs	2	2	X-ray	Partly on the bladder wall and bladder stone	Cystoscopy—suprapubic cystotomy	Favorable
Atakan [34], 2002	27	Pelvic pain, frequency, dysuria	8 yrs	2	3	X-ray	Posterior bladder wall and bladder stone	Cystoscopy—suprapubic cystotomy	Favorable
Mahmutyazicioğlu [35], 2002	30	Pelvic pain, dysuria, frequency	10 yrs	2	NS	US, X-ray	The left side of the anterior bladder wall	Cystoscopy	Favorable
Senanayake [36], 2002	22	Pelvic pain, vaginal pain	6 yrs	2	NS	X-ray, US	Bladder stone	Cystoscopy, extraperitoneal cystotomy	Favorable
Dabbas [37], 2002	32	Left loin pain, frequency, dysuria, hematuria	5 m	2	NS	US, X-ray, IVU	Right ureteric orifice with minimal hydronephrosis, omentum/bowel	Laparoscopy, suprapubic cystotomy	Favorable
Demirci [38], 2003	33	Irritative lower UTS	2 yrs	2	4.1	X-ray	Bladder stone	Cystoscopy—fragmented with a lithotripter	Favorable
Wei [39], 2003	74	Frequency, dysuria, urge incontinence	35 yrs	1	4.5	US, X-ray	Partly on the bladder wall, with bladder stone	Cystoscopy	Favorable
Rafique [40], 2003	32	Lower UTS	NS	2	NS	X-ray, US	Intravesical migration with secondary stone formation	Suprapubic cystostomy	Favorable
Eke [41], 2003	31	Missing strings	3 wks	NS	NS	US	Bladder wall	Laparoscopy, open cystostomy	Favorable
Eskandar [42], 2003	23	Suprapubic pain	34 m	2	NS	US	Right lateral trigone of the bladder	Cystoscopy	Favorable
Nwofor [43], 2003	44	Frequency, dysuria	10 yrs	2	4 and 2	US, X-ray	Posterior bladder wall, with stone; bladder—uterine fistula	Cystolithotomy	Favorable
Ozçelik [44], 2003	28	Recurrent UTI, pelvic pain	6 m	2	NS	US, X-ray	Dome of the bladder, bladder stone	Cystoscopy, laparoscopy	Favorable
Ozgür [45], 2004	31	Hematuria, frequency, urge incontinence, dysuria	7 yrs	2	5.5	X-ray, CT	Bladder stone	Cystolithotomy	Favorable
Hick [46], 2004	31	Recurrent UTI, irritative voiding symptoms	NS	2	8	X-ray	Bladder stone	Cystolithotomy with Burch colposuspension	Favorable
Tunçay [47], 2004	30	Suprapubic pain	5 yrs	2	NS	US, cystoscopy	Posterior bladder wall, with bladder stone	Cystoscopy, laparotomy—bladder repair	Favorable
Zafar [48], 2004	34	Recurrent UTS	NS	2	NS	X-ray	One IUD in a bladder stone, and one in the posterior bladder wall	Cystoscopy	Favorable
Dede [49], 2006	28	Recurrent UTI	6 yrs	2	NS	X-ray, US	Partly at the fund uteri, omentum, bowel, posterior wall of the bladder	Laparoscopy—incompletely removed; cystoscopy—IUD + stone removed	Favorable
Hoşcan [50], 2006	29	Pelvic pain, frequency	8 yrs	5	NS	X-ray, US	Posterior bladder wall	Cystoscopy—suprapubic cystotomy	Favorable
Gillis [51], 2006	28	Suprapubic pain, recurrent UTS, hematuria	5 yrs	2	NS	X-ray, CT	Bladder stone	Cystoscopy—complete extraction	Favorable
Khan [52], 2006	28	Frequency, suprapubic discomfort, UTI	18 m	3	NS	US	LNG-IUS on the posterior bladder wall and bladder stone	Cystoscopy—fragmented with a lithotripter	Favorable
Singh [53], 2007	30	Pelvic pain, dysuria, gross hematuria	3 yrs	2	NS	X-ray, US	The posterior wall of the bladder and bladder stone	Cystoscopy—fragmented with a lithotripter	Favorable
Nouira [54], 2007	42	Pelvic pain, dysuria	10 yrs	2	NS	X-ray	Bladder stone	Cystoscopy—fragmented with a lithotripter	Favorable
	37	Irritative voiding symptoms, recurrent UTI	5 yrs	2	NS	X-ray, IVU	Bladder stone	Cystoscopy—complete extraction	
	38	Recurrent UTI, hematuria	9 yrs	2	NS	IVU	Bladder stone	Cystoscopy—fragmented with a lithotripter	
	30	Dysuria, hematuria	11 yrs	2	3	X-ray, IVU	Bladder stone	Cystoscopy—fragmented with a lithotripter	
	26	Recurrent UTI	6 yrs	2	NS	X-ray, US, IVU	Bladder stone	Cystoscopy—complete extraction	
	40	Pelvic pain	10 yrs	2	1	US	Bladder stone	Cystoscopy—complete extraction	
Istanbulluoglu [55], 2008	32	Turbid urine, recurrent UTI	6 yrs	2	2.5	X-ray, US	Right wall of the bladder and bladder stone	Cystoscopy—fragmented with a lithotripter	Favorable
Rafique [56], 2008	28	Recurrent UTI	5 yrs	NS	NS	X-ray, US	Bladder stone	Cystoscopy—litholapaxy	Favorable
	32	Hematuria	5 yrs	NS	NS	X-ray, US	Bladder wall and bladder stone	Open cystotomy	
	35	Frequency, dysuria	3 yrs	NS	NS	X-ray, US	Bladder wall and bladder stone	Open cystotomy	
	40	Hematuria, dysuria	2 yrs	NS	NS	X-ray, US	Bladder stone	Cystoscopy—litholapaxy	
El-Hefnawy [57], 2008	24	Lower UTS	6 m	2	NS	X-ray, US, CT	Bladder stone	Cystoscopy	Favorable
	27	Lower UTS	1 yr	2	NS	X-ray, US, CT	Bladder stone	Cystoscopy—litholapaxy	
	20	Lower UTS	2 yrs	2	NS	X-ray, US, CT	Bladder stone	Cystoscopy—litholapaxy	
	28	Lower UTS	1 wk	2	NS	X-ray, US, CT	Vesicouterine fistula	Open repair of the fistula	
	32	Lower UTS	1 yr	2	NS	X-ray, US, CT	Bladder wall and bladder stone	Cystoscopy—litholapaxy	
	25	Lower UTS	14 m	2	NS	X-ray, US, CT	Bladder stone	Cystoscopy—litholapaxy	
	29	Right loin pain	2 yrs	2	NS	X-ray, US, CT, IVU	Right hydroureteronephrosis	Open ureteroneocystostomy	
	38	Right loin pain	15 m	2	NS	X-ray, US, CT, IVU	Right hydroureteronephrosis	Open ureteroneocystostomy	
Yensel [58], 2009	30	Dysuria	2 yrs	2	NS	US	Bladder wall	Cystoscopy	Favorable
Mustafa [59], 2009	46	Suprapubic pain	12 yrs	2	3	X-ray, CT	Bladder wall and bladder stone	Cystoscopy—fragmented with a lithotripter	Favorable
Chuang [60], 2010	28	Suprapubic pain, frequency	3 yrs	2	0.9	US; CT	Dome of the bladder, bladder stone	Cystoscopy, laparoscopy	Favorable
Vural [61], 2010	48	Pelvic pain	15 yrs	2	NS	US	Dome of the bladder, bladder stone	Cystoscopy, laparoscopy	Favorable
Karsmakers [62], 2010	74	Weakness, dyspnea, abdominal discomfort	39 yrs	1	NS	X-ray, cystoscopy	Posterior bladder, above the trigone, and bladder stone, vesicovaginal fistula	Vaginal lithotomy, without cystolithotomy and fistula repair	Hemo-dialysis
Al-Awadi [63], 2011	57	Dysuria, lower abdominal symptoms, recurrent UTI	25 yrs	2	NS	CT, cystoscopy	Bladder wall and bladder stone	Cystolithotomy	Favorable
Dar [64], 2011	60	Suprapubic pain	20 yrs	NS	8	US, X-ray	Bladder stone	Cystolithotomy	Favorable
Ko [65], 2011	50	Frequency, dysuria, hematuria	20 yrs	2	NS	US, X-ray	Bladder wall and bladder stone	Cystoscopy—fragmented with a lithotripter	Favorable
	26	Hematuria, dysuria	NS	NS	NS	X-ray	Bladder wall and bladder stone near the urethra	Cystoscopy	
Chae [66], 2012	45	Dysuria, frequency, suprapubic pain	10 yrs	3	4	X-ray, CT	Bladder stone	Cystoscopy	Favorable
Shin [67], 2012	38	Recurrent UTI, dysuria, chronic pelvic pain	12 yrs	2	1.9	X-ray, CT urography	Posterolateral bladder wall and bladder stone	Cystoscopy, laparoscopy	Favorable
Jeje [68], 2012	45	Gross hematuria, irritative lower UTS	20 yrs	NA	NS	US, X-ray	Posterior bladder wall and bladder stone	Cystoscopy, suprapubic cystostomy	Favorable
Guner [69], 2013	41	Recurrent dysuria, frequency, hematuria	15 yrs	2	NS	US, X-ray, CT	Partially penetrated to the bladder wall	Cystoscopy	Favorable
Ebner [70], 2013	47	Dysuria, hypermenorrhagia	4 wks	3	NS	US, X-ray	Freely movable LNG-IUP	Cystoscopy	Favorable
Campobasso [71], 2014	39	Recurrent UTI, dysuria	5 yrs	2	NS	US, CT urography	Dome of the bladder	Cystoscopy, laparoscopy	Favorable
Gunbey [1], 2014	46	Pelvic pain	NS	2	NS	US (5 wks pregnancy)	The cervix and the bladder lumen	Cystoscopy, TOP	Favorable
Scovell [72], 2014	50	Abdominal pain, recurrent UTI	20 yrs	2	2.6	CT	Posterolateral bladder wall and bladder stone	Cystourethroscopy—cystolitholapaxy	Favorable
Simsek [73], 2014	62 1/7	Dysuria, hematuria, recurrent UTI	NS	NS	NS	X-ray, US	Bladder wall and bladder stone	Cystoscopy—fragmented with a lithotripter	Favorable
Voulgaris [74], 2015	30	Dysuria, hematuria, recurrent UTI	1 yr	NS	NS	US	Right lateral bladder wall and bladder stone	Cystoscopy	Favorable
Kart [75], 2015	44	Lower UTS	15 yrs	NS	NS	US, X-ray	Posterior bladder wall and bladder stone	Cystoscopy—fragmented with a holmium laser lithotripter	Favorable
Liu [76], 2015	35	Irritative voiding symptoms, gross hematuria	10 yrs	5	NS	X-ray, CT	Posterior bladder wall and bladder stone	Cystoscopy, laparoscopy—IUD removed	Favorable
Niu [77], 2015	29	Lower UTS	NS	NS	NS	US (early pregnancy)	Partial perforation of the bladder	Cystoscopy	Favorable
Bashir [78], 2016	60	Calculus formation	12 yrs	2	NS	US, X-ray	IUD migrated to the bladder	Cystolithotomy	Favorable
Shen [79], 2016	42	Gross hematuria, urinary urgency, dyspareunia	NS	NS	NS	US	Bladder stone	Cystoscopy—fragmented using a holmium laser cystolitholapaxy	Favorable
Gyasi-Sarpong [80], 2016	33	Dysuria, abdominal pain, frequency	3 yrs	2	NS	US	Bladder stone	IUD removal by cystoscopy	Favorable
Jin [81], 2016	43	Recurrent suprapubic pain, dysuria, frequency	20 yrs	2	NS	US, CT, hysteroscopy (uterine IUD removed)	Second IUD in the posterior wall of the bladder, bladder stone, and omentum	Cystoscopy, laparoscopy—IUD removed	Favorable
Chai [82], 2017	26	Frequency, abdominal pain for 5 yrs, hematuria	6 yr	2	NS	Plain X-ray, cystoscopy	IUD migrated to the bladder	IUD removal by cystoscopy	Favorable
De Silva [83], 2017	48	Lower UTS	15 yrs	2&5	6 × 5	US	Bladder stone	Vesicolithotomy	Favorable
Clancy [84], 2017	45	Suprapubic pain, frequency	1 yr	3	NS	US	Extrinsic bladder mass	Cystoscopy	Favorable
Wang [85], 2017	39	Abdominal pain	12 yrs	NS	NS	US, X-ray, IVU	Ureteral wall, with bladder stone (uro-nephrosis)	Ureteroscopy—pneumatic lithotripsy, ureteral stent	Favorable
	25	Frequency, dysuria, hematuria	2 yrs	NS	2 × 2.5	US, IVU, CT	Posterior superior bladder wall and bladder stone	Cystoscopy—pneumatic lithotripsy, laparoscopy	
	41	Right hydronephrosis	NS	NS	NS	IVU, CT	Stricture and proximal expansion in the right lower ureter	Laparoscopy—removed IUD, ureter reconstruction, ureteral stent	
Cheung [5], 2018	34	Fever	NS	NS	NS	US, CT, cystoscopy	The anterior surface of the bladder	Laparotomy—bladder repaired	Favorable
Vahdat [86], 2019	31	Lower UTS	8 yrs	2	NS	X-ray, US, hystero-salpingraphy	Partly in the bladder lumen and in the bladder wall	IUD removal by cystoscopy	Favorable
Li [87], 2019	30	Left side lumbago	5 yrs	NS	NS	CT (hydro-nephrosis, ureterectasis)	Near the left lower ureter	Integrant ureterectomy by retro-peritoneoscopy and laparoscopy	Left nephrectomy
Niu [88], 2019	57	Suprapubic pain	26 yrs	2	1	US, X-ray	One limb in the uterine cavity, one in the bladder, embedded in a bladder stone	Hysteroscopy, cystoscopy—using a holmium laser lithotripter	Favorable
Jievaltiene [89], 2019	41	Uterine contractions, pelvic pain	18 m	2	NS	US (40 wks pregnancy)	Anterior wall of the bladder	Remove the IUD during the cesarean	Favorable
Basiri [90], 2019	37	Recurrent UTI, dysuria	11 yrs	2	0.1	US, X-ray	Bladder wall and bladder stone	Cystoscopic extraction	Favorable
Zhang [91], 2019	38	Recurrent urinary urgency, dysuria	11 yrs	2	NS	US, CT	Bladder wall with extensive benign hyperplasia	Transurethral bladder resection of granuloma	Favorable
Zhang [92], 2020	39	Frequency	2 m	NS	NS	CT, cystoscopy	Bladder wall and uterine wall	Combined hysteroscopy + cystoscopy to repair bladder and uterus	Favorable
Dappa [93], 2020	55	Hydronephrosis, history of cervical carcinoma + CRT	NS	NS	1.8 & 4.5	CT	Bladder stone	Cystoscopy—mechanical lithotripsy	Vesicovaginal fistula
Badu-Peprah [94], 2020	27	Mild pelvic pain	2 yrs	NS	NS	US, X-ray, CT	Bladder wall and bowel (ileum about 17 cm from the ileocecal junction)	Laparotomy (bowel and serosa of the bladder were repaired)	Favorable
Christodoulides [95], 2020	67	Recurrent UTI, dysuria, pelvic pain	20 yrs	NS	NS	US	Free-floating intravesical stone	Cystolitholapaxy	Favorable
Benaguida [96], 2021	27	Pelvic pain with minimal metrorrhagia	8 m	NS	NS	Abdominal-pelvic CT	Anterior peritoneal collection above the bladder	Laparoscopy—incision/aspiration of the collection IUD removed	Favorable
Lin [97], 2021	51	Intermitent pink urine	10 yrs	2	NS	CT, cystoscopy	Extrauterine location, with a long tail, penetrated the bladder	Laparoscopy—repair the bladder	Favorable
Yang [98], 2021	42	Asymptomatic (desire to conceive)	18 yrs	NS	NS	US, X-ray	Partial perforation of the bladder	Hysteroscopy, single-incision laparoscopy	Favorable
Liu [99], 2021	37	Lower UTS, frequency, hematuria	9 yrs	NS	NS	US, CT	Right bladder wall and bladder stone	Cystoscopy—cystotomy with IUD removal	Favorable
	46	Frequency, dysuria	10 yrs	NS	NS	CT	Near the left ureteral orifice, with stone on its surface	Cystoscopy—cystotomy with IUD removal	
Akhtar [100], 2021	40	Asymptomatic	NS	2	NS	US	Right parametrium and right lateral bladder wall	Laparoscopy was converted into laparotomy; removed IUD and bladder wall repaired	Favorable
	35	Dysuria	1 yr	NS	1	US (3 m pregnant), CE-CT	Anterior bladder wall	Elective laparotomy 6 m after C-section, partial cystectomy	
Han [101], 2021	40	Asymptomatic	NS	NS	NS	CT	Bladder wall	Cystoscopy	Favorable
Qu [102], 2021	30	Dysuria, ocassional gross hematuria	8 yrs	NS	NS	US, X-ray, CT	Dome of the bladder and bladder stone	Cystoscopy, laparoscopic exploration	Favorable
Salih [103], 2022	38	Gross hematuria, abdominal pain	15 yrs	2	NS	US, CT, cystoscopy	Posterior bladder wall	Cystoscopy, open trans-vesical surgery—bladder wall repaired	Favorable
Ago [104], 2022	36	Pelvic pain, dysuria, hematuria	10 yrs	NS	7	US, X-ray	Bladder stone	Open vesicolithotomy	Favorable
Agarwal [105], 2022	55	Recurrent UTI	NS	NS	NS	X-ray	Bladder stone	Cystoscopy—cystolithotripsy	Favorable
Moy [106], 2022	46	Hematuria, frequency	13 yrs	2	NS	CT urogram, cystourethroscopy	One arm—right postero-lateral bladder wall, second arm intra-peritoneally	Robotic Da Vinci—assisted laparoscopic cystostomy	Favorable
Al-Khatlan [107], 2023	29	Missing strings	NS	2	NS	Abdominal—pelvic X-ray, CT, laparoscopy	Intraperitoneally, adhesions between the bladder dome and omentum	Laparoscopy—removed IUD from the omental/bladder adhesions	Favorable
Adeyanju [108], 2023	44	Pelvic pain, hematuria	7 yrs	2	NS	US, X-ray	Bladder stone	Laparotomy—urinary dome incision	Favorable
Saputra [109], 2023	30	NS	NS	NS	NS	US, X-ray	Bladder stone	Cystoscopy	Favorable
Agil [110], 2024	36	Lower UTS	6 yrs	2	NS	CT	Posterolateral rectovesical fistula, bladder stone	Cystoscopy—lithotripsy	Rectovesi-cal fistula
Our case, 2023	55	Asymptomatic during investigations for lumbar discopathy	19 yrs	2	NS	X-ray, US, CT scan, hysteroscopy/laparoscopy	Submucosa of the bladder; pelvic mass of adhesions between omentum, sigmoid, bladder	Laparoscopic IUD removal after adhesiolysis and cystorrhaphy	Favorable

UTI—urinary tract infections, UTS—urinary tract symptoms, IVU—intravenous urogram, CRT—chemoradiotherapy, NS—not specified, wks—weeks, m—months, yrs—years.

## Data Availability

The data presented in the study are included in the article/Supplementary Material, further inquiries can be directed to the corresponding authors.

## Supplementary Materials

The following supporting information can be downloaded at https://www.mdpi.com/article/10.3390/jcm13144233/s1: Table S1. Representation of the risk of bias of the case reports we analyzed in this review.
