# Supplementary material for: Intrauterine Contraceptive Device Migrated in the Urinary Tract: Case Report and Extensive Literature Review
Source: J Clin Med. 2024 Jul 19;13(14):4233. doi: 10.3390/jcm13144233 (PMC11278257; doi:10.3390/jcm13144233)
Supplement: Supplementary file 1 [file jcm-13-04233-s001.zip › jcm-3051768-supplementary.pdf]

Table S1. Representation of the risk of bias of the case reports we analyzed in this review.

	Does the patient represent the whole investigator experience, or is the selection method unclear to the extent that other patients with similar presentation may not have been reported?	Was the exposure adequately ascertained?	Was the outcome adequately ascertained?	Were other alternative causes that may explain the observation ruled out?	Was there a challenge/ rechallenge phenomenon?	Was there a dose–response effect?	Was follow-up long enough for outcomes to occur?	Is the case described with sufficient details to allow other investigators to replicate the research or to allow practitioners to make inferences related to their own practice?
Woods, 1980	Green	Green	Green	Green	Green	Green	Green	Green
Sasidharan, 1988	Green	Green	Green	Green	Green	Green	Green	Yellow
Khan, 1990	Green	Green	Green	Green	Green	Green	Green	Green
Dietrick, 1992	Green	Green	Green	Green	Green	Green	Green	Green
Shratter, 1993	Green	Yellow	Green	Green	Green	Green	Green	Red
el-Diasty, 1993	Green	Green	Green	Green	Green	Green	Green	Green
Caspi, 1996	Green	Green	Green	Green	Green	Green	Green	Yellow
Bjørnerem, 1997	Green	Green	Green	Green	Green	Green	Green	Green
Szabó, 1997	Green	Green	Green	Green	Green	Green	Green	Yellow
Maskey, 1997	Green	Green	Green	Green	Green	Green	Green	Yellow
	Green	Green	Green	Green	Green	Green	Green	Yellow
Cumming, 1997	Green	Green	Green	Green	Green	Green	Green	Green
Yalçin, 1998	Green	Green	Green	Green	Green	Green	Green	Green
Olaore, 1999	Green	Green	Green	Green	Green	Green	Green	Yellow
Sehgal, 2000	Green	Green	Green	Green	Green	Green	Green	Green
Güvel, 2001	Green	Green	Green	Green	Green	Green	Green	Green
Atakan, 2002	Green	Green	Green	Green	Green	Green	Green	Green
Mahmutyazicioğlu, 2002	Green	Green	Green	Green	Green	Green	Green	Green
Senanayake, 2002	Green	Green	Green	Green	Green	Green	Green	Yellow
Dabbas, 2002	Green	Green	Green	Green	Green	Green	Green	Green
Demirci, 2003	Green	Green	Green	Green	Green	Green	Green	Green
Wei, 2003	Green	Green	Green	Green	Green	Green	Green	Green
Rafique, 2003	Green	Yellow	Green	Green	Green	Green	Green	Red
Eke, 2003	Green	Green	Green	Green	Green	Green	Green	Yellow
Eskandar, 2003	Green	Green	Green	Green	Green	Green	Green	Green
Nwofor, 2003	Green	Green	Green	Green	Green	Green	Green	Green
Ozçelik, 2003	Green	Green	Green	Green	Green	Green	Green	Yellow
Ozgür, 2004	Green	Green	Green	Green	Green	Green	Green	Green
Hick, 2004	Green	Green	Green	Green	Green	Green	Green	Red
Tunçay, 2004	Green	Green	Green	Green	Green	Green	Green	Red
Zafar, 2004	Green	Yellow	Green	Green	Green	Green	Green	Red
Dede, 2006	Green	Green	Green	Green	Green	Green	Green	Green
Hoşcan, 2006	Green	Green	Green	Green	Green	Green	Green	Green
Gillis, 2006	Green	Green	Green	Green	Green	Green	Green	Yellow
Khan, 2006	Green	Green	Green	Green	Green	Green	Green	Yellow
Singh, 2007	Green	Green	Green	Green	Green	Green	Green	Yellow
Nouira, 2007	Green	Green	Green	Green	Green	Green	Green	Yellow
	Green	Green	Green	Green	Green	Green	Green	Yellow
	Green	Green	Green	Green	Green	Green	Green	Yellow
	Green	Green	Green	Green	Green	Green	Green	Green
	Green	Green	Green	Green	Green	Green	Green	Yellow
	Green	Green	Green	Green	Green	Green	Green	Green
Istanbulluoglu, 2008	Green	Green	Green	Green	Green	Green	Green	Green
Rafique, 2008	Green	Yellow	Green	Green	Green	Green	Green	Yellow
	Green	Yellow	Green	Green	Green	Green	Green	Yellow
	Green	Yellow	Green	Green	Green	Green	Green	Yellow
	Green	Yellow	Green	Green	Green	Green	Green	Yellow
El-Hefnawy, 2008	Green	Green	Green	Green	Green	Green	Green	Yellow
	Green	Green	Green	Green	Green	Green	Green	Yellow
	Green	Green	Green	Green	Green	Green	Green	Yellow
	Green	Green	Green	Green	Green	Green	Green	Green
	Green	Green	Green	Green	Green	Green	Green	Yellow
	Green	Green	Green	Green	Green	Green	Green	Yellow
	Green	Green	Green	Green	Green	Green	Green	Green
	Green	Green	Green	Green	Green	Green	Green	Green
Yensel, 2009	Green	Green	Green	Green	Green	Green	Green	Green
Mustafa, 2009	Green	Green	Green	Green	Green	Green	Green	Green
Chuang, 2010	Green	Green	Green	Green	Green	Green	Green	Green
Vural, 2010	Green	Green	Green	Green	Green	Green	Green	Yellow
Karsmakers, 2010	Green	Green	Green	Green	Green	Green	Green	Yellow
Al-Awadi, 2011	Green	Green	Green	Green	Green	Green	Green	Yellow
Dar, 2011	Green	Green	Green	Green	Green	Green	Green	Yellow
Ko, 2011	Green	Green	Green	Green	Green	Green	Green	Yellow
	Green	Yellow	Green	Green	Green	Green	Green	Red
Chae, 2012	Green	Green	Green	Green	Green	Green	Green	Green
Shin, 2012	Green	Green	Green	Green	Green	Green	Green	Green
Jeje, 2012	Green	Yellow	Green	Green	Green	Green	Green	Yellow
Guner, 2013	Green	Green	Green	Green	Green	Green	Green	Green
Ebner, 2013	Green	Green	Green	Green	Green	Green	Green	Green
Campobasso, 2014	Green	Green	Green	Green	Green	Green	Green	Green
Gunbey, 2014	Green	Yellow	Green	Green	Green	Green	Green	Red
Scovell, 2014	Green	Green	Green	Green	Green	Green	Green	Green
Simsek, 2014	Green	Yellow	Green	Green	Green	Green	Green	Red
Voulgaris, 2015	Green	Yellow	Green	Green	Green	Green	Green	Yellow
Kart, 2015	Green	Yellow	Green	Green	Green	Green	Green	Yellow
Liu, 2015	Green	Green	Green	Green	Green	Green	Green	Yellow
Niu, 2015	Green	Yellow	Green	Green	Green	Green	Green	Red
Bashir, 2016	Green	Green	Green	Green	Green	Green	Green	Green
Shen, 2016	Green	Yellow	Green	Green	Green	Green	Green	Red
Gyasi-Sarpong, 2016	Green	Green	Green	Green	Green	Green	Green	Yellow
Jin, 2016	Green	Green	Green	Green	Green	Green	Green	Yellow

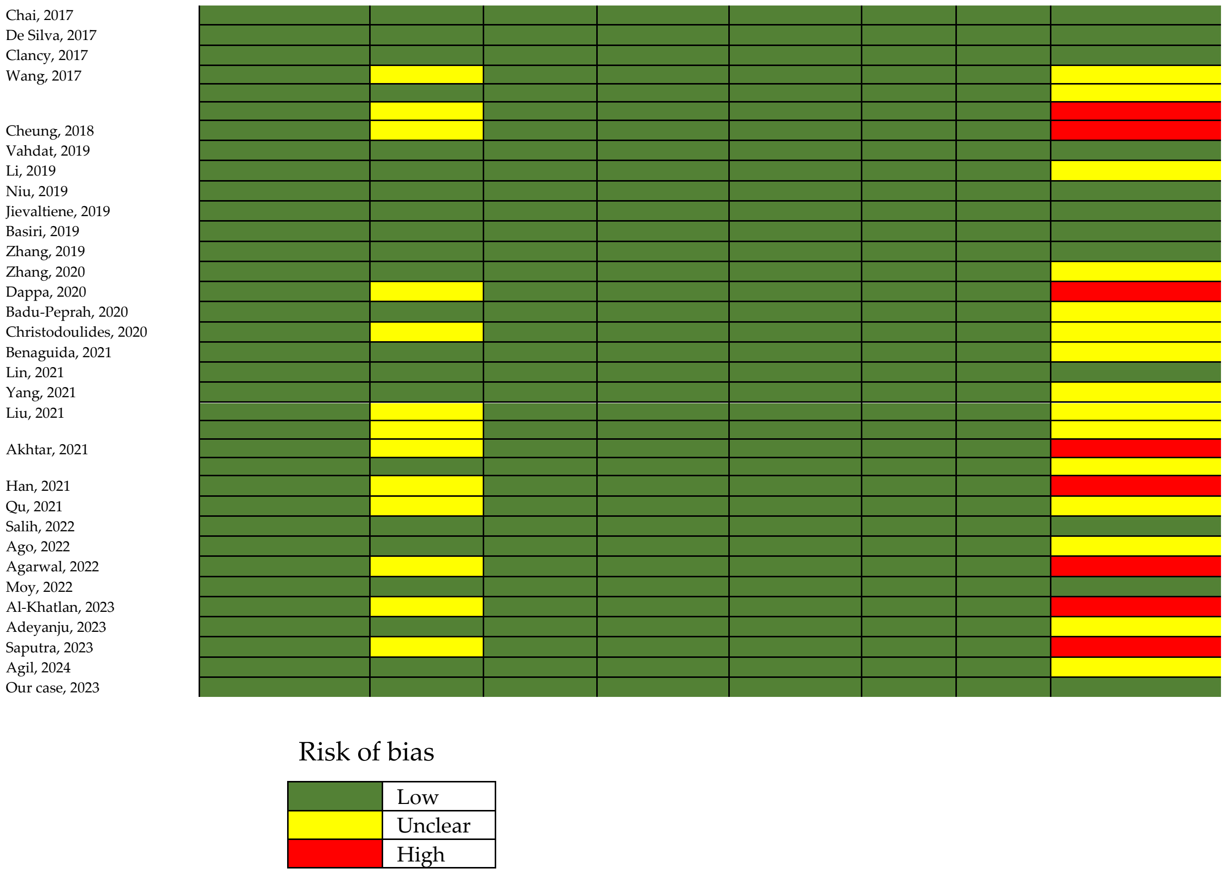
Chai, 2017
De Silva, 2017
Clancy, 2017
Wang, 2017
Cheung, 2018
Vahdat, 2019
Li, 2019
Niu, 2019
Jievaltiene, 2019
Basiri, 2019
Zhang, 2019
Zhang, 2020
Dappa, 2020
Badu-Peprah, 2020
Christodoulides, 2020
Benaguida, 2021
Lin, 2021
Yang, 2021
Liu, 2021
Akhtar, 2021
Han, 2021
Qu, 2021
Salih, 2022
Ago, 2022
Agarwal, 2022
Moy, 2022
Al-Khatlan, 2023
Adeyanju, 2023
Saputra, 2023
Agil, 2024
Our case, 2023
Risk of bias
Low
Unclear
High
